# Dynamic Networks from Hierarchical Bayesian Graph Clustering

**DOI:** 10.1371/journal.pone.0008118

**Published:** 2010-01-11

**Authors:** Yongjin Park, Cristopher Moore, Joel S. Bader

**Affiliations:** 1 Department of Biomedical Engineering and High-Throughput Biology Center, Johns Hopkins University, Baltimore, Maryland, United States of America; 2 Department of Computer Science and Department of Physics, University of New Mexico, Albuquerque, New Mexico, United States of America; 3 Santa Fe Institute, Santa Fe, New Mexico, United States of America; Center for Genomic Regulation, Spain

## Abstract

Biological networks change dynamically as protein components are synthesized and degraded. Understanding the time-dependence and, in a multicellular organism, tissue-dependence of a network leads to insight beyond a view that collapses time-varying interactions into a single static map. Conventional algorithms are limited to analyzing evolving networks by reducing them to a series of unrelated snapshots.

Here we introduce an approach that groups proteins according to shared interaction patterns through a dynamical hierarchical stochastic block model. Protein membership in a block is permitted to evolve as interaction patterns shift over time and space, representing the spatial organization of cell types in a multicellular organism. The spatiotemporal evolution of the protein components are inferred from transcript profiles, using *Arabidopsis* root development (5 tissues, 3 temporal stages) as an example.

The new model requires essentially no parameter tuning, out-performs existing snapshot-based methods, identifies protein modules recruited to specific cell types and developmental stages, and could have broad application to social networks and other similar dynamic systems.

## Introduction

Systems biology suggests that we can understand a biological system by decomposing it hierarchically into modular sub-systems. In a molecular-scale network, these sub-systems include multi-molecular complexes that form dynamic associations with other complexes. These systems can be represented naturally as time-dependent networks whose vertices are biomolecules (DNA/genes, RNA/transcripts, proteins, metabolites) and whose edges represent physical interactions.

Large-scale compendiums of physical interactions are primarily static lists that lack the dynamic aspects of living molecular systems. Protein-protein interactions make up by far the largest interaction class available in compendiums. These interactions come primarily from high-throughput screens that may not be specific to a single temporal stage (such as affinity purification/mass spectrometry of yeast protein complexes obtained as an average over the cell cycle) or may involve an engineered system entirely removed from natural cellular dynamics (such as two-hybrid screens). Other interactions inferred from numerous bioinformatics methods, including cross-species inference, necessarily lack information about spatiotemporal network dynamics.

The approach used here is to assume that interactions collected in a compendium represent a superposition of the possible interactions that could occur within a cell. From a different data source, we obtain a spatiotemporal profile of the active network components. These data sets are joined in a probabilistic model, termed a dynamic hierarchical stochastic block model, to infer network evolution. Our application is to protein interaction networks, but the same techniques could be applied to other types of networks, or to a complex network of multiple interaction types. Spatiotemporal dynamics of proteins are inferred from transcript presence or absence in mRNA profiling studies, an admittedly inaccurate proxy for protein levels but nevertheless the primary type of dynamic data readily available for cellular systems.

The application is to dynamic evolution of protein networks required for root development in *Arabidopsis*, based on a classic data set generated by Benfey and coworkers [1]. The physical interactions used in this study are obtained from work by Geisler, Provart and coworkers [2] and available in The Arabidopsis Information Resource (TAIR) ftp://ftp.arabidopsis.org/home/tair/Proteins/ [3].

This work, termed DYHM for “Dynamic Hierarchical Model”, builds on previous studies that used mRNA abundance as a proxy for protein abundance, and when applied to yeast cell cycle data [4] showed the existence of protein complexes that are specific to cell cycle phases [5], [6]. These studies, however, typically consider each temporal stage as an independent snapshot. A protein complex at an initial time has no explicit connection with itself at subsequent times. Analysis of a series of snapshots becomes idiosyncratic and *ad hoc*, both in terms of the algorithms for clustering a snapshot of a network (often by single-linkage clustering with an adjustable threshold on the confidence of each network edge) and in following the evolution of a complex across snapshots.

A further assumption of previous methods, and of this work, is that an interaction will occur if interacting components are both expressed. In other words, if an interaction between proteins A and B is reported in a database, and transcripts corresponding to genes A and B are present, then the interaction is assumed to be active. In reality, interactions can depend on protein modifications, localization changes, co-expression of other proteins, and environmental cues. Our model does not address these difficult points.

To solve the problem of network dynamics, we adapt a probabilistic generative model that has performed exceptionally well for analyzing static networks. The model is termed a stochastic block model, which in our context means that we assign proteins to blocks (or groups), and the probability of an interaction pattern between two proteins depends only on the groups to which they are assigned.

Recent work showed that hierarchical block models, which represent intermediate levels of organization in a network, provide state-of-the-art performance in identifying meaningful groups and predicting missing links [7], [8]. Vertices in an observed network are assigned to leaf nodes in a hierarchically branching tree. We introduce an extension in which group-group interactions are constant over space and time, but group membership can vary dynamically. Dynamic evolution of group-group interaction parameters can be added to this model (see Discusion).

As a second independent contribution, we have made this model scalable to larger networks by replacing slowly-converging Markov chain Monte Carlo (MCMC) sampling with the variational solution to a mean-field approximation. The mean-field problem can be solved in polynomial time, compared with the complex optimization of the original problem thought by many to be NP-hard with an exponentially large search space. The mean-field approximation converges rapidly and accurately for synthetic data, and provides new biological insight when applied to root development.

For static networks, related work has used Variational Expectation-Maximization [9] to identify interacting communities in interaction networks [10]. This previous work assumed a homogeneous pattern of interactions both within groups and between groups, as opposed to the heterogeneity observed in biological networks. Another type of static network model, solvable using expectation-maximization [11], uses an asymmetric model in which groups of vertices interact with individual vertices [12]–[14]. This latter model has very recently been extended to dynamic networks [15].

## Results

Our dynamic network clustering algorithm has essentially two adjustable parameters: (1) the number of clusters, defined by the branching depth 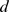 of a hierarchical tree; (2) the relative importance given to optimizing clusters within each snapshot compared to enforcing smoothness between snapshots, defined by a parameter 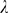. Methods that sample over different numbers of leaf nodes are possible [10]. In practice, we have found that results for occupied leaf nodes are stable provided that some leaf nodes are unoccupied (see Methods).

The second parameter, 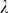, interpolates between an independent model for each snapshot (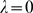) and a single model that superimposes all the snapshots (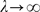). As discussed below, however, a value of 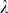 can in fact be selected using a penalized likelihood. Results are presented first for simulated data, to establish the performance of the method, and then for *Arabidopsis* root development.

### Simulation Studies

#### Static synthetic data

Prior to testing on dynamic networks, we tested our hierarchical model on static networks, comparing the variational approximation to the original MCMC algorithm and to competing methods for analyzing interaction networks. We selected two representative competing methods, the popular MCODE [16] that extracts clusters from locally dense regions, and the hypergeometric p-value for neighbor sharing that ranks pairs of vertices without an intermediate step of predicting clusters or complexes [17].

We assessed performance from predicted pairwise co-membership scores. Overall tests were repeated for 100 different static networks, and the precision and recall were computed according to amassed counts of false-positives, false-negatives, and true-positives. The number of groups within each simulated network was selected uniformly from 5 through 10 inclusive, and the number of vertices within each group was also selected uniformly from 5 through 10. The probability Pwithin of within-group edges was selected uniformly between 0.05 and 0.1, and the probability Pbetween of between-group edges was selected uniformly between 0.05 and 0.08. Parameter sets with Pwithin 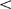 Pbetween were discarded. We then generated a random network from the parameters, knowing true membership of all vertices. After ranking pairs by each method, we constructed Precision-Recall (PR) curves.

#### Performance on static networks

**Figure 1:**
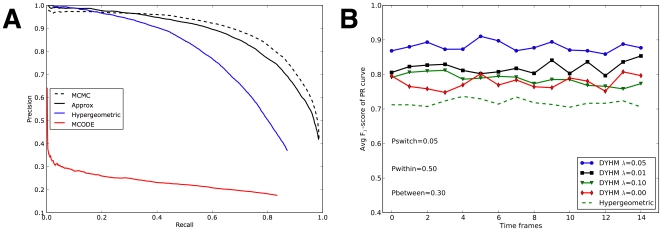
Simulation study. **(A)** Comparison on static synthetic networks. From top to bottom, lines correspond Precision-Recall curves of four different methods. *Dashed black*: Hierarchical model trained by MCMC sampling. *Solid black*: Hierarchical model trained by variational approximation. *Solid blue*: Hypergeometric method [17]. *Solid red*: MCODE [16]. **(B)** Comparison on dynamic synthetic networks. From top to bottom, lines denote correspond to F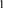 scores over time frames. *Blue circle*: DYHM with 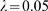. *Black squre*: DYHM with 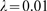. *Green triangle*: DYHM with 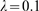. *Red diamond*: DYHM with 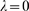. *Dashed green*: Hypergeometric method [17] applied separately to each each time frame.

While the other methods rely on local metrics, inference on the hierarchical model seeks to optimize a total configuration of vertex membership. In our results (Fig. 1A), both the MCMC and the variational approximation for the hierarchical model are far superior to other methods tested. The poor outcome of MCODE may arise from its greedy local search strategy. Once a misleading “seed” vertex is chosen, incorrect clustering may be locked in.

The MCMC algorithm, which samples from a full joint distribution, performs somewhat better than the variational approximation in which all group memberships are decoupled (Fig. 1A, black solid line versus dashed line). The drawback of MCMC, however, is the long computational time to obtain converged results. The variational method, in contrast, takes polynomial time, and it converged quickly for all the networks we tested. We found that the variational approach was at least 10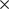 faster for these small simulated networks, and for larger networks (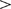
100 vertices) we did not have sufficient CPU resources to test the MCMC algorithm.

#### Dynamic synthetic data

The dynamic data was generated by assigning 30 total vertices initially to 5 groups. A snapshot of a set of edges was then generated by adding within-group edges to the snapshot with probability Pwithin, and adding between-group edges with probability Pbetween. After each snapshot, the edges are erased, each vertex switches to a different group at random with probability Pswitch, and the process continues. This process permits the number of vertices in each group to change with time. The known group assignments provide a gold standard of known positives to assess the inferred co-membership probabilities.

Results from DYHM using a depth-3 hierarchy (8 groups) at various values of 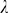, including extreme values corresponding to independent and superimposed snapshots, were compared with co-membership inferred by the hypergeometric method ([17]; see Methods). For each snapshot we generated a PR curve and a corresponding F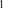 score (the maximum harmonic mean of precision and recall along the curve).

#### Performance on dynamic networks

On relatively easy data sets (Pwithin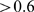 and Pbetween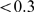), all models work well (results not shown). On harder simulation tests, however, DYHM gave superior performance. An example is Pwithin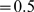, Pbetween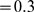, and Pswitch = 0.05 (Fig. 1B). The value of 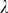 selected by penalized likelihood (which requires no knowledge of the true group assignments) also gives the best performance in predicting time-dependent co-membership, F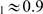 corresponding to roughly 90% precision and recall. It performs better than independent analysis of each static snapshot, corresponding to 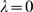, with F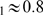. We note that the 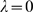 version of DYHM itself out-performs the hypergeometric predictor, which gives F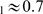.

We further tested the ability of 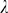 to track networks with increasingly labile group membership, ramping Pswitch through values 0.01, 0.05, 0.2, 0.3, and 0.5, on non-trivially simulated network data with Pwithin and Pbetween respectively fixed at 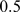 and 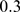. In all cases tested, the value of 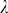 with the best penalized likelihood gave the best performance (results not shown).

### Arabidopsis Root Development

#### Dynamic biological network

**Figure 2:**
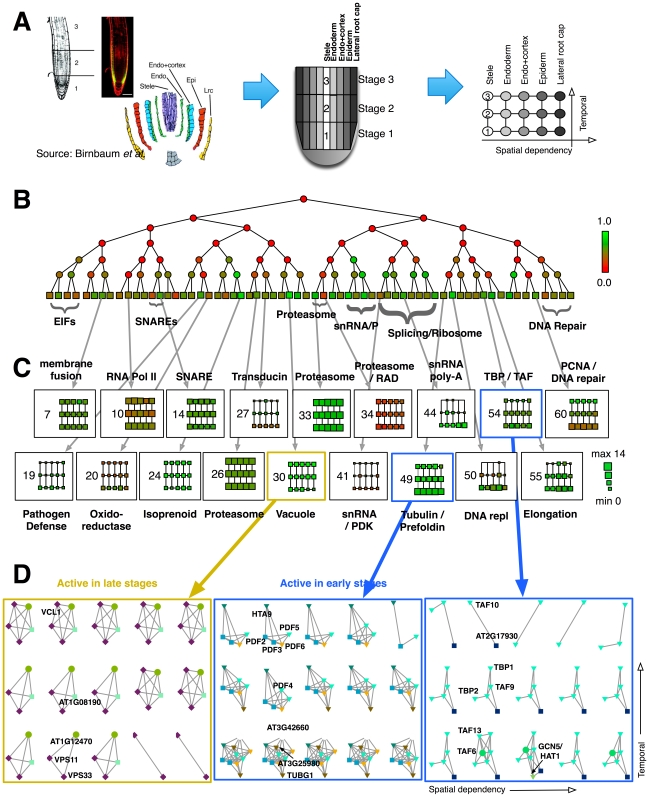
Arabidopsis root development. **(A)** Lateral root sections correspond to distinct tissues, and vertical sections correspond to to distinct developmental stages. **(B)** Average hierarchical decomposition of 15 networks. Node color indicates enrichment (green) or depletion (red) of within-cluster (at terminal nodes) or between-cluster (at internal nodes) edges relative to random connectivity. **(C)** The evolution of each cluster is displayed over the 5 tissues and 3 stages. Size indicates the number of proteins within the cluster, and color indicates edge enrichment. **(D)** Selected micro-views on network dynamics. The leftmost example shows delayed activity of two genes in developmental process. The other two examples include complexes that are more active at early stages. Sub-networks in each panel were drawn in identical topology. Gene names are labeled once. See text for details of selected clusters.

The root is an ideal model for development because temporally staged samples are easily obtained by cutting further back from the root tip, and distinct cell and tissue types are observed radially outward from the root center (Fig. 2A). A classic study mapped gene expression activity in 5 spatial regions across 3 developmental stages [1], yielding 15 spatiotemporal snapshots.

High-confidence interactions for the corresponding proteins (confidence value 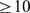) were extracted from TAIR Interactome 2.0 [2]. For this superposition of all genes active anywhere in the root map, we iteratively deleted network vertices with degree less than or equal to 3 until no more vertices could be removed. The resulting network had 332 vertices and 1163 edges. Subnetworks were then generated by extracting the active genes (expression level 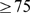 as reported by [1]; see Discussion) and their interactions for each of the 15 snapshots. Each snapshot had approximately 150 to 220 genes and 5 interactions per gene (Table. 1).

**Table 1:** The spatiotemporal variation of active subnetworks.

	Stele	Endoderm	Endo + Cortex	Epiderm	Lateral root cap
Stage 3	217 (569)	215 (565)	225 (603)	219 (586)	211 (543)
Stage 2	182 (415)	185 (432)	193 (462)	188 (440)	172 (391)
Stage 1	150 (328)	151 (331)	156 (354)	144 (324)	135 (285)

The numbers of active genes at each position are shown without parentheses; the numbers of active interactions are shown within the parentheses.

#### Model selection

The depth of the hierarchical tree was set to 6 (64 groups). Results for occupied groups were substantially unchanged for depth-7 trees (128 groups, results not shown). DYHM introduces 8 spatiotemporal couplings with strength 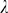 for adjacent tissues and stages (Fig. 2A). For the observed data 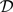 and a specific value of 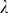, we used a penalized likelihood to determine the degree of time-smoothness:
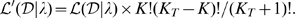
With 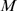 total groups (here 64), a total of 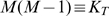 directed transitions are possible. Of these, a subset 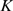 are observed at least once across the 8 coupled snapshots. The penalty 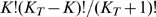 gives equal weight to each of the 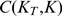 models with exactly 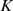 transitions, which results in a steeper penalty for models with more transitions. This penalty arises from a Bayesian viewpoint in which each of the 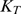 possible transitions is observed independently with probability 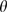. Integrating 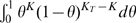 produces the stated form of the penalized likelihood. We performed a search over a sparse grid, 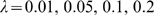, and selected 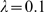 as the optimal value.

#### Hierarchical clustering and spatiotemporal mapping

Dynamical clustering using DYHM produces hierarchical cluster assignments for each of the 15 spatiotemporal samples. A reduced view of the results, averaging the inferred memberships over the 15 samples, is provided (Fig. 2B). The node color represents the averaged interaction enrichment. Leaf nodes, shaped as squares, are groups of clustered genes. These leaves are indexed from 1 (leftmost) to 64 (rightmost) for later reference. Zoomed-in views below illustrate how selected clusters evolve over space and time in increasing resolution (Fig. 2C,D).

This tree view shows that most of the groups are assortative (green nodes, enriched for self-interactions), which is typical of protein complexes. Some leaf nodes assemble hierarchically into larger assortative modules, and these components often share similar biological functions. For instance, four of small nuclear RNA/RNP complexes (snRNA/P) are located adjacently and form a clade (terminal leaves #39-40). Cladistic assignments are also observed for EIF (eukaryotic translation initiation factor) complexes (leaves #1-4) and Splicing/Ribosome complexes (leaves #41-48).

An overview of terminal groups shows how each of the 64 clusters varies over the 15 spatiotemporal snapshots in terms of occupancy and within-cluster interactions (Fig. 2C). Several of the clusters correspond to protein complexes that appear constitutively active, whose transcripts would typically be filtered out as unchanging. Examples are #7 (membrane fusion), #10 (RNA Pol II), #14 (syntaxin and SNARE proteins), and #26 and #33 (proteasome). A more dynamic pattern is observed for clusters that are conditionally activated, most often with complex members present at early times and then absent at later times to yield a smaller core complex. Examples are #44 (mRNA polyadenylation), #49 (a core of prefoldin and the H2A.Z histone variant HTA9 has additional tubulin-related complex members during stage 1), and #60 (a PCNA DNA repair complex is present in stage 1 but vanishes in stages 2 and 3). These observations are consistent with the inference from mRNA data of rapid mitotic activity during stage 1 [1].

#### TATA box-binding protein complex

A detailed view of cluster #54, involved in transcription from TATA box promoters, highlights this pattern of dynamic complex membership (rightmost of Fig. 2D). TATA box-binding protein associated factors (TAFs) have time-specific and tissue-specific activity [18]. One member of the TAF family, TAF10 (aka AT4G31720, TFIID15), has preferential and transient expression during the middle developmental stages of plant organs. Disrupting this tight regulation causes pleiotropic phenotypic changes and abnormal morphologies [18].

The majority of the genes in cluster #54 are TAFs, including TAFII15/TAF10, TAFII21/TAF9, and TAFII59/TAF6. In the root expression map, TAF10 is a core member of this complex, while other members are transient. Along the temporal axis, the TAF10-TAF9-TFIID-1 complex is present during early root development, persists partially through stage 2, and in the mature root only TAFII15, TBP2, and the uncharacterized PIK-related kinase AT2G17930 remain. TAFs provide DNA-binding specificity for TFIIDs, which bind to the basal transcriptional machinery [19]. The TAF6 (TAFII59) protein appears to be present primarily in stage 1, although absent from the stele. This factor has a core interaction motif required for H3/H4 heterodimerization [19], which suggests regional epigenetic modification in early development. At the early stage, this complex also has HAT1 as a member, a histoneacetyltransferase that is a positive regulator of transcription in root morphogenesis.

## Discussion

We have presented a new method for modeling the spatiotemporal dynamics of a biological network. The model takes as input a series of discrete network states coupled in space and time and infers a structure of dynamic groups that enter and leave the network, possibly merging or separating from existing groups.

When applied to synthetic data, the model performs substantially better than existing methods that consider each network snapshot in isolation. It uses a variational approach that is much faster than previous Monte Carlo methods and is scalable to genome-sized networks.

Applied to a biological data set obtained from *Arabidopsis* root development, the model reveals the dynamic organization of network components. Previous analysis of this mRNA data set was limited to time-varying and spatially-varying genes. Of the roughly 22,000 transcripts interrogated, 1/2 were not expressed in the root, 1/4 showed differential regulation over space and time, and 1/4 were expressed constitutively. These unchanging transcripts are filtered out by traditional gene expression analysis.

For our analysis, the activity of each network component is inferred from transcript profiling, and the set of possible interactions is obtained from a database compendium. Our dynamic network model reveals that the constitutive components form the core of complexes that evolve through the addition and subtraction of dynamic modules. We are also able to observe modules that are strictly limited to specific spatiotemporal states and vanish elsewhere.

Converting real-valued gene expression levels to a binary presence/absence score for a protein is admittedly problematic. First, protein levels do not necessarily track mRNA levels. Second, the level of protein activity may not be adequately represented by a binary 0/1 score. We adopted this approach in part because it was used in the original study. Given the promising performance of our initial application, further work may benefit by incorporating quantitative measures of gene or protein activity.

Our model considers only about 5% of the 10,000 genes expressed in *Arabidopsis* root because these are the only ones with high-confidence interaction data. Access to a greater number of interactions, for example including medium and low-confidence interactions, will help retain more genes in the network model. The method can also be generalized to incorporate edge confidence scores. The model is readily extended to incorporate additional types of network edges, such as gene regulatory interactions inferred from ChIP/chip experiments for *Arabidopsis* [20] and other species.

The model we have introduced can be readily generalized to incorporate other time-dependent edge types, such as protein-DNA regulatory interactions or protein-protein modifications. Time dependence in the model described is limited to time-varying module membership, but patterns of module-module interaction are held constant. As an analogy, consider a model of a citation network where patterns of citation by an author depend on the author's research group. In this model, a graduate student will follow the pattern of his or her PhD mentor, and then will take on the pattern of his or her postdoctoral mentor. The patterns of the mentors' groups remain fixed, however. In a more general model, the pattern for each mentor can itself evolve. This more general model is also amenable to an efficient variational optimization.

These methods may have significant applications to other types of time-varying networks, such as social networks or other dynamic social groups where interaction are recorded over time and space.

## Methods

### Probabilistic Model

#### Definition

Given network data 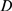 consisting of a set of vertices 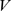 and an adjacency matrix 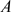 (or a set of edges), Clauset *et al.* [7], [8] suggested a model that hierarchically decomposes this set of vertices. The model likelihood is expressed as a product of Bernoulli distributions from iteratively dividing 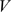 into “left” and “right” subgroups. These divisions take place at internal nodes of a binary dendrogram. More formally, each internal node 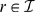 splits graph vertices assigned to it into left 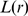 and right 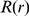 subsets. The likelihood is written in terms of the relative edge 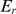 and non-edge counts 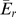 between these left and right subsets, which are the sufficient statistics of a Bernoulli distribution parameterized by edge probability 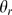. We rewrite this as follows:
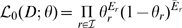



The tree-based decomposition need not be conducted to completion, with each leaf having only a single graph vertex. Rather we establish a fixed tree depth, and allow the very bottom nodes, which we call terminal nodes or leaves, to have more than one graph vertex. The terminals take the same form of the Bernoulli likelihood, this time counting within-group edges for the graph vertices assigned to each terminal leaf node. Within-group edge probabilities are described by parameters 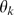 for each 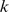 in the total terminal set 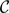. The extended likelihood is
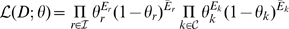
where 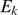 and 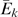 respectively denote the counts of edges and non-edges among the vertices under the 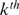 terminal node. The model is readily extended to heterogeneous independent data sets, 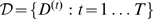, as 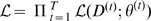. Note that the parameters 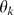 can be integrated out in a Bayesian setting, yielding no adjustable parameters other than tree depth.

#### Intractability

The maximum likelihood estimation of the optimal tree (the optimal assignment of graph vertices to terminal leaves) is challenging since it involves learning most likely left-right divisions for each parameter estimation task. The problem is similar to learning evolutionary parameters from an unknown phylogenetic tree structure. Related phylogeny algorithms escape this obstacle by performing Bayesian model averaging rather than attempting to identify the optimal model. For example, the Metropolis-Hastings algorithm [21] can sample plausible tree structures according to the likelihood; then, based on the ensemble of these trees, evolutionary parameters such as mutation rates can be estimated [22]. The previous works of Clauset *et al.* [7], [8] uses model averaging by sampling over trees with probabilities obtained from maximum likelihood parameter estimates.

In practice, this strategy is suitable for moderately small networks, and the model asymptotically converges to the Gibbs distribution of probable hierarchical structures, with probability proportional to their likelihood. Unfortunately, convergence can be difficult to determine, and adequate sampling can require substantial CPU resources for even moderately sized networks (100 to 1000+ vertices).

#### Structural approximation

To achieve scalability on a large biological network, we modified the original algorithm in two ways: fixed tree structure and variational approximation. Here we fix the depth of the terminals, and the dendrogram structure is a perfect binary tree. Each terminal node of the tree represents a group of zero or more vertices from the original graph. This structural assumption not only brings about a fixed probabilistic framework, which suits a variational approximation, but also reduces the search space from 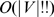 to 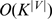, where 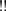 is the double factorial, 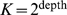 is the number of terminal nodes, and 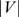 is the cardinality of network vertices. As described in the results, this fixed dendrogram does not appear to change the results for occupied terminals provided that the tree is sufficiently deep, which is readily tested by runs at multiple tree depths.

For an explicit model definition, 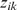 is a latent variable indicating whether vertex 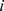 is assigned to the terminal node 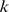: 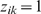 only if 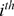 vertex is assigned to that node, otherwise 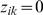. Using this, the sufficient statistics of the internal edge and non-edge counts are
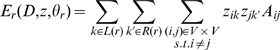


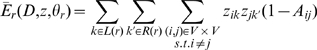
and those of the terminals are
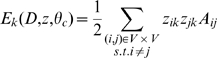


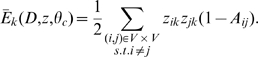
For succinctness, we also define the following potential functions for the log-likelihood of the internals and terminals.
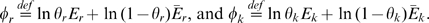
Combining all these, the total likelihood with the flat priors of the parameters becomes
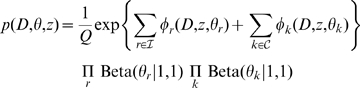
(1)where 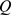 is a normalizing constant. We use standard non-informative priors for the Beta distribution. The inference is now on the latent variables and the parameters; we may solve this by exploiting Jensen's inequality
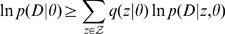
(2)where 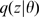 is a distribution over the latent variables, and 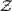 denotes the overall space. If the posterior computation for 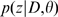 is readily available, setting 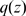 to this probability will give an improved lower bound as in generalized expectation-maximization [11]. This method is equivalent to Gibbs-Bogoliubov-Feynman variational mean field theory.

#### Variational approximation

In our model, the space of latent variables 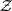 can expand exponentially to 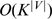 due to the dependency of the variables (in an undirected probabilistic graphical model, the structure is simply a clique). One easy solution is to sample according to the total likelihood score over this space of 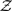. We in fact have tested this MCMC algorithm along with the following variational approximations. But this necessitates the second approximation. Here we use a variational approximation posing a slightly different bound where we also take care of the uncertainty of 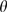 [23]:
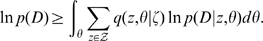
(3)Then the inference task consists of finding 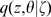 with respect to some variational parameters 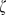, tightening the lower bound. Maximizing the lower bound is equivalent to minimizing the Kullback-Leibler (KL) divergence 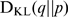 [23]. By minimizing the KL divergence we characterize the distribution of 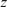 and 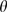 approximately. The detailed steps are provided below as update equations.

Now, let us extend this further to an ordered series of observed networks, 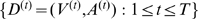, whose vertex sets 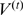 and adjacency matrices 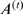 are dynamic. But we additionally believe that an abrupt change between 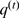 and 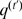 is rare when times 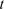 and 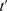 are adjacent. Note also that the index 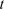 is more general than a sequential time index, and we think more generally of the set of snapshots 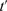 that are neighbors of snapshot 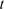. So, we consider this divergence as well in the following objective function:
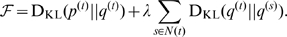
(4)The first term provides a conventional mean-field approximation between a true model distribution 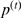 and the surrogate factorized 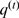, and the second handles our belief in spatiotemporal smoothness. In other words, we want to find 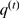 as close as possible to 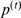, but not very apart from the neighboring snapshots 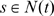. We call our novel approach a Dynamic Hierarchical Model (DYHM).

We note again that despite the complicated looking model structure, there is in fact only one adjustable parameter, 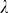, which controls the spatiotemporal smoothness. Setting 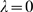 is equivalent to treating the snapshots as if they were independent, and large 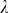 gives static group membership. The remaining parameters are all optimized as part of the model and are not subject to tuning. Furthermore, the model likelihood can be used as a guide for selecting 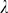 itself, leading to a model with no adjustable parameters, other than the depth selected for the hierarchical tree.

### Time-Constrained Mean-Field Approximation

First let us define each term of Eq. 4. To pose a tractable inference problem, we represent the joint probability density (Eq. 1) as a factorized mean-field distribution
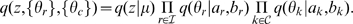
(5)Each factored distribution is defined by the variational parameters, 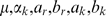,
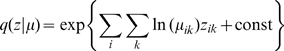


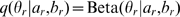


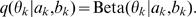
Then, the hard combinatorial problem can converted to a tractable optimization problem. Here, we minimize two Kullback-Leibler distances: (Eq. 6) divergence of the approximate surrogate from the true distribution, and (Eq. 7) divergence between distributions at adjacent time frames:
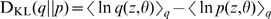
(6)

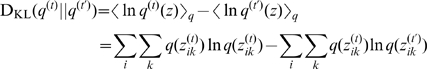
(7)where 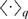 denotes an expectation taken with respect to the surrogate distribution of time 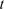, i.e., 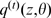. Thanks to the convexity of the KL-divergence, we are guaranteed to reach a local optimum by setting the first derivatives to zero. We iteratively optimize each variational parameter until convergence.

#### Latent variable update

The expected values of the latent group assignments, 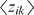, correspond to the 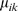 parameters in the variational distribution (Eq. 5). For algebraic convenience, we account for time-dependency among active genes by introducing auxiliary variables: let 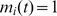 indicate that gene 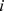 is active at time 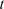, and 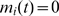 if inactive. We can then rewrite the objective function of the update of 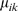 as follows:
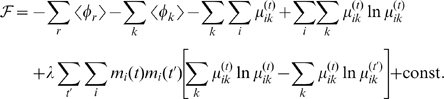
Introducing the Lagrangian 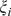 to take care of a constraint, 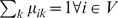, the derivative is
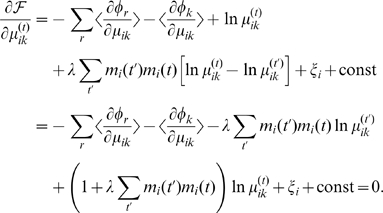
To be more explicit, the derivatives of the potential functions are
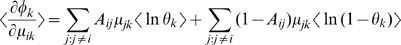


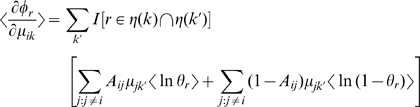
where 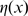 denotes a set of a terminal 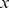
's ancestry, and 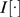 is an indicator function. The update equation is simply
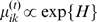
(8)where
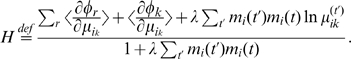
From the above, we can consider two extreme cases:
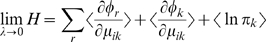


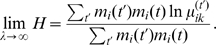
The first assumes independence between time points, while the latter approximates the current position by the geometric mean of adjacent ones.

#### Update for the tree parameter

Given the latent variable assignments, more precisely their expected assignments 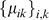, we can optimize the tree parameters by taking the derivative with respect to 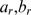 for all 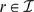, and 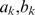 for all 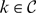. The updates for internal nodes 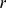 are 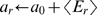 and 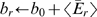, using the expected edge and non-edge counts for the left and right subsets of the internal nodes. We use priors 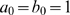, corresponding to the non-informative priors of Eq. 1. The parameters of the potential functions are
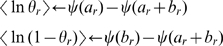
(9)where 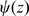 is the digamma function, 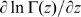. Likewise, the parameters for the terminal nodes can be updated: we set for all 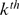 terminal nodes, 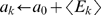, 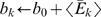, and
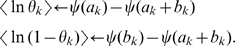
(10)


#### Overall algorithm

Starting from a randomly initialized 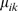 for all 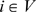 and 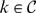, we update the tree parameters according to Eq. 9 and Eq. 10, and then approximate 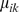 according to Eq. 8. Theses two steps are repeated until convergence. In practice, we ran the algorithm multiple times with 7 random restarts and generally observed similar variational likelihoods and similar group structures. Results are provided for the best likelihood over the random restarts.

### Co-Membership Scores

The co-membership probability of two different vertices 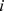 and 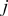 is computed from the 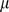 parameters trained in Eq. 8. The probability of these vertices being co-clustered is
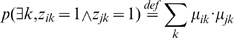
where we do not consider the special case 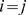. Note unlike the original MCMC algorithm [7], we only need to compute these metrics once at the final converged parameter values.

### Tree Depth

The depth of the tree is a fixed parameter in the variational algorithm (whereas in the original MCMC method the tree depth changes dynamically during the sampling). As part of our method for an input network, we ran the variational algorithm for a series of increasingly deep trees. In practice, the variational solution for a tree of depth 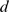 can be used as the starting point for the next simulation of depth 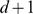, but we did not do so. For simulated input where the number of groups is known, we found that trees that were sufficiently deep usually sorted each group into its own terminal node, with the remaining terminal nodes unoccupied. Results for co-membership were then stable as the tree depth increased further, the main difference being more unoccupied terminal nodes and greater computational time (results not shown). We used the observation of unoccupied terminal nodes as a metric for selecting sufficiently deep trees for biological data sets. All the reported results are essentially unchanged for deeper trees.

### Comparison to Other Methods

#### MCMC

Exploiting the conjugacy between the Beta and binomial distributions, an analytical derivation of 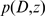 of Eq. 3 is straightforward. As an alternative to the variational approximation, a stochastic simulation via MCMC gives the asymptotically correct distribution of 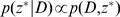. While sampling according to this distribution, we collect the co-membership scores. We can summarize them by taking an average. This provides a direct comparison to the variational approximation.

#### Hypergeometric method and MCODE

The hypergeometric method followed Goldberg and coworkers [17] using the hypergeomtric distribution to calculate the p-value for shared neighbors of two network vertices. MCODE is the work of Bader and Hogue [16]. We gradually changed the cutoff value defining clusters to examine all pairwise co-membership scores.

### Precision-Recall

We used a precision-recall curve, and its summary F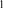 score, to assess the quality of the scores produced by the tested methods. They are defined as
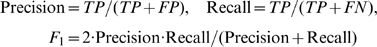
where 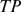, 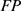, and 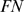 are the number of true positives, false positives, and false negatives.

### Availability

Space limitations prevent full presentation of results. Source code (BSD open source license) and a complete catalog of protein complexes are available from the authors, http://www.baderzone.org/, and as Source Code and Dataset S1.

## Supporting Information

Source Code and Dataset S1 DYHM source code and datasets.
(0.25 MB TAR)
